# Whooping Cough

**Published:** 1875-01

**Authors:** 


WHOOPING COUGH.—Dr. Wilde claims that he can cure every case of whooping cough within eight days, by the following mode of treatment:

The patient should be kept in-door, to avoid exposure to cold. Then, at the commencement of every paroxysm, a teaspoonful of the following mixture:

| | |
|---|---|
| ℞.—Chloroformi, . . . . . . . | f ℥ i. |
| Æther. Sulphur., . . . . . . . | f ℥ ij. |
| Ol. Terebinth., . . . . . . . | f ℥ iij. |

M.—Is poured on a cloth and held about two inches from the mouth of the patient until the paroxysm subsides.—*Med. Ex.*

"WELL, DOCTOR," said a chap just from the dentist's chair, "how much do you ax for the job? Guy! but you did it quick, though!" "My terms," replied the dentist, "are one dollar." "A dollar for half a minit's work! one dollar—thunder! Why, a doctor down t' our place drawed a tooth for me two years ago, and it took him two hours. He dragged me all around the room, and lost his grip half a dozen times. I never seed such hard work. And he only charged me twenty-five cents. A dollar for a minit's work! you must be jokin'."—*Pharmacal Gazette.*

CHARLOTTE CUSHMAN says: "I found life sadly real and intensely earnest; and, in my ignorance of other ways of study, I resolved to take therefrom my text and my watchword. To be thoroughly in earnest, intensely in earnest, in all my thoughts and in all my actions—whether in my profession or out of it—became my one single idea, and I honestly believe herein lies the secret of my success in life. I do not believe that great success in any art can be achieved without it."

